# Object Recognition and Dorsal Stream Vulnerabilities in Children With Early Brain Damage

**DOI:** 10.3389/fnhum.2022.733055

**Published:** 2022-05-12

**Authors:** Ymie J. van der Zee, Peter L. J. Stiers, Heleen M. Evenhuis

**Affiliations:** ^1^Royal Dutch Visio, Rotterdam, Netherlands; ^2^Department of General Practice, Intellectual Disability Medicine, Erasmus MC, Rotterdam, Netherlands; ^3^Department of Neuropsychology and Psychopharmacology, Maastricht University, Maastricht, Netherlands

**Keywords:** L94, visual search, visual attention, motion perception, object recognition, dorsal stream dysfunction, visuomotor skills, early brain damage

## Abstract

**Aim:**

Visual functions of the dorsal stream are considered vulnerable in children with early brain damage. Considering the recognition of objects in suboptimal representations a dorsal stream dysfunction, we examined whether children with early brain damage and impaired object recognition had either general or selective dorsal stream dysfunctions.

**Method:**

In a group of children with early brain damage (*n* = 48) we evaluated the dorsal stream functioning. To determine whether these patients had an increased risk of a dorsal stream dysfunction we compared the percentage of patients with impaired object recognition, assessed with the L94, with the estimated base rate. Then we evaluated the performance levels on motion perception, visual attention and visuomotor tasks in patients with (*n* = 18) and without (*n* = 11) object recognition abnormalities. A general dorsal stream dysfunction was considered present if a patient showed at least one abnormally low score in two out of three additional dorsal stream functions.

**Results:**

Six of the eighteen (33.3%) patients with object recognition problems scored abnormally low on at least two additional dorsal stream functions. This was significantly higher than the base rate (*p* = 0.01). The difference of 24.1% between the patients with and without object recognition problems was not significant. Of the patients with object recognition problems 72.2% had at least 1 dorsal weakness, whereas this was only the case in 27.3% of patients without object recognition problems. Compared to patients with normal object recognition, patients with object recognition problems scored significantly more abnormally low on motion perception and visual attention (*p*s = 0.03) but did not differ on visuomotor skills.

**Conclusion:**

Children with object recognition problems seem at risk for other dorsal stream dysfunctions, but dysfunctions might be rather specific than general. Multiple functions/aspects should be evaluated in neuropsychological assessment of children at risk.

## Introduction

Studies on visual perception in children with developmental disorders (Atkinson et al., 2003) and early brain damage (Gunn et al., 2002; Fazzi et al., 2004) suggest that the dorsal stream of the cerebral visual system is more vulnerable than the ventral stream. The dorsal stream is associated with motion perception (Braddick et al., 2001; Stiers et al., 2006), visuomotor integration (James et al., 2003), and visual attention (Pollmann et al., 2003). Different brain areas are thought to be crucial for these functions: V5/MT + for global motion, motion speed and motion-defined form perception (Ho et al., 2005; Reiss et al., 2005; Wang et al., 2007); the superior temporal sulcus (STS) area for biological motion perception (Bonda et al., 1996) and target detection (Pollmann et al., 2000); the temporoparietal junction (TPJ) for attention shift (Corbetta and Shulman, 2002), the intraparietal sulcus (IPS) for visuospatial attention (Corbetta and Shulman, 2002) and response preparation (Pollmann et al., 2000); the inferior parietal lobule (IPL) for visuomotor planning (Glover, 2004), and the superior parietal lobule (SPL) for attentional bias (Pollmann et al., 2003), action, visuomotor control (Glover, 2004). The ventral stream, which projects into the inferotemporal cortex, is associated with form perception, object recognition (Atkinson et al., 2003; James et al., 2003) and face recognition (Atkinson et al., 2003).

Although object recognition is primarily considered a ventral stream function, existing evidence indicates that it is not a ventral stream function alone: IPS (Vuilleumier et al., 2002; Konen and Kastner, 2008), IPL (Vuilleumier et al., 2002; Eger et al., 2007) and SPL (Eger et al., 2007) play a role in object recognition in suboptimal representations. Damage in the parietal lobe is associated with impaired recognition in the following suboptimal representations: incomplete pictures (Renzi and Spinnler, 1966; Warrington and James, 1967), overlapping drawings (Warrington and Taylor, 1973; Fazzi et al., 2004), objects presented from an unconventional view (Warrington and Taylor, 1973; Vaina, 1994), and drawings degraded by noise (Warrington and Taylor, 1973).

Studies on dorsal stream functions in children with early brain damage or developmental disorders indicate that different aspects of object recognition in suboptimal representations (Stiers et al., 2001; Ortibus et al., 2009) and motion perception can be selectively impaired (Gunn et al., 2002; Ho et al., 2005; MacKay et al., 2005; Mendes et al., 2005; Reiss et al., 2005; Jakobson et al., 2006; Wang et al., 2007). In most studies single functions or aspects were studied, therefore it is unknown how often multiple dorsal stream functions are affected. One study in prematurely born children with complications such as periventricular white matter disease and retinopathy of prematurity, showed that 20–55% of the patients had clinically significant impairments in different dorsal stream functions (Jakobson et al., 2006). However, it was not reported whether individual patients were impaired on multiple tasks, and object recognition in suboptimal representations was not addressed in this study.

The aim of the present study was to determine whether other dorsal stream dysfunctions are commonly present in children with an identified dorsal stream dysfunction. In this study, the L94 was used to detect children with a dorsal stream dysfunction in a group of children with (indications of) early brain damage. The L94 is a valid diagnostic test battery for object recognition in suboptimal representations. Abnormally low scores on the L94 are associated with parietal lobe damage (Ortibus et al., 2009). We hypothesized that patients with object recognition abnormalities show significantly more often abnormally low scores on other dorsal stream aspects, i.e., motion perception, visual attention, and visuomotor skills, than children with normal object recognition. We also examined how often a general dorsal stream dysfunction was present, i.e., how often at least 2 out of three additional dorsal stream functions were weak.

## Materials and Methods

### Participants

The patient group consisted of 48 children at risk for object recognition problems, because of brain damage, indications of brain damage, and/or reports of suspicion of visual perceptual impairments mentioned in their medical records. Patients were recruited through rehabilitation centers in the Rotterdam area (Rijndam Rehabilitation Center and Royal Dutch Visio, Center of Expertise for blind and partially sighted people, *n* = 19) and through the Laboratory of Neuropsychology at the University Hospital in Leuven, Belgium (*n* = 29). Their chronological age ranged from 4 y1m to 14y7m (*M* = 7y3m, *SD* = 2y4m).

Studies were approved by the Ethics Committees of the Erasmus Medical Center MEC-2006-056) and the Catholic University of Leuven. Informed consent was obtained for all participants through their parents or guardians.

### Procedures

#### Medical History and Orthoptic Assessment

Data on etiology of the brain damage and imaging results (CT and/or MRI), gestational age and recent orthoptic assessments were gathered from available medical records. The participant was invited for an orthoptic assessment, if no recent orthoptic assessment was done. Eye movements, visual acuity with up-to-date refractive corrections (lenses or glasses), visual field, and binocular vision were assessed by trained professionals (orthoptists) using (developmental) age-appropriate tests. Visual field was mainly assessed with the confrontation visual field exam (Donder’s test).

Participants wore their prescribed glasses or lenses during the developmental age and dorsal stream assessment.

#### Developmental Age Estimation

To control for cognitive impairments in the patient group, we estimated the participant’s developmental age (DA). The developmental age was defined as the median age-equivalent of multiple subtests measuring performance IQ (PIQ) (Stiers et al., 2001): the patient’s raw scores on non-verbal subtest were converted to age-equivalents, then the median was calculated, resulting in the developmental age at the time of IQ-assessment (DA_IQ_). We corrected for a time-lag between IQ assessment and dorsal stream function assessment using the following formula: DA_dorsal_ = (DA_IQ_/CA_IQ_)*CA_dorsal_, where CA stands for chronological age.

To minimize the burden on the patients we decided to use recent intelligence results when available. If not available, we only studied non-verbal intelligence, because only non-verbal cognitive ability, and not verbal cognitive skill, is predictive of perceptual performance (Ito et al., 1996, 1997; Stiers et al., 1999). Although the use of a single intelligence test is preferable, the cognitive consequences of the brain damage and the broad age range in the patient group made this impossible. Because of the strong correlations (0.79–0.93) between the Performance IQs of different intelligence tests (SON-R IQ, WPSSI-R, WISC-R, WISC-III) (Moore et al., 1998; Oosterbaan et al., 2006), we considered these tests interchangeable for the DA_*IQ*_ estimation.

#### Dorsal Stream Function Assessment

The following dorsal stream functions were studied in arbitrary order: object recognition in suboptimal representations; motion perception; visual attention; visuomotor skills. Published or preliminary reference data was used to classify performance levels. We used DA, instead of CA, as entry to the reference tables. In case PIQ ≥ 100, DA exceeded CA, we used the patient’s CA as entry to the reference tables.

If DA was out of range of the norm tables, we used the nearest age group. An object recognition score below the 5^th^ percentile were defined as an abnormal performance. For other function tasks, a score below the 10^th^ percentile was defined as abnormally low.

Testing was done by trained senior psychology students and neuropsychologists. All computerized tasks were run on a laptop connected to a 15-inch CRT monitor. Participants with refractive errors wore their prescribed glasses and were placed in front of the screen at approximately 40 cm.

#### L94: Object Recognition

To assess object recognition in suboptimal representations we used five computerized subtasks of the L94 (Stiers et al., 1998, 2001) that require recognition of line drawings of everyday objects: Visual matching (VISM); drawings occluded by noise (NOISE); overlapping line drawings (OVERL); unconventional object views (VIEW); De Vos (DE VOS).

##### Visual Matching

This task consists of 1 example and 10 items: line drawings of everyday objects in prototypical view. Each item is presented for 1 s, followed by a screen with a semantically identical object and three distracters. The participant must point out the target object. Items scores are 1 (correct) or 0 (incorrect).

##### Drawings Occluded by Noise

This task consists of 1 example and 6 items. Each object is presented for 2 s and is partly occluded by noise. Participants must name or describe the presented object. Noise level decreases until the object is recognized correctly. There are 7 noise levels (60, 50, 42, 36, 29, 24, and 0%). 0% noise level is considered the control condition. Item score is (7-j)/(7-1), where j is the number of noise levels presented before the participant recognized the object.

##### Overlapping Line Drawings

This task consists of 1 example and 6 items. Items are presented for 6 s and consist of two, three of four overlapping objects, followed by the target objects and two distracters, all presented separately. Participants must point out the target objects. The level of overlap decreases until all target objects are indicated correctly. There are four levels of overlap: full overlap, partially overlap, touching, and separate presentation. The last condition is considered the control condition. Item score is (4-j)/(4-1), where j is the number of overlap levels presented before a correct response is given.

##### Unconventional Object Views

This task consists of 20 items. Items are presented for 3 s. Half of the items are presented in three conditions: Unconventional view, less unconventional view, and conventional view. For the other half of the pictures, the level of unconventional view decreases to the conventional view in four conditions. The level of conventional view decreases until the participants named or described the object correctly. The conventional view is considered the control condition. Items scores is (k—j)/(k—1), where k is the total number of conditions and j is the number of conditions presented before the participant recognized the object.

##### De Vos

This task consists of 43 items. There is no time constraint. Items are presented in a target condition and a control condition. In the target condition objects are less easy to recognize because they are embedded in context, they are partial drawn, only contours are presented, a typical part of the object is omitted, or they are presented in an unconventional view. Participants must name or describe the target object. Item score is 1 (recognized) or 0 (not recognized in target condition).

Performance was expressed in a subtask score. The subtask score was the average item score, with exclusion of items not recognized in the control condition. Items with an incorrect response in the control condition were indicated as inconclusive and excluded, because an incorrect response could not only be the result of a recognition problem, but also of other problems (language etc.).

We excluded the items rifle, bench, and alarm clock from the analysis of VIEW, because Dutch controls tended to name these objects differently, for example by their general category and often did not change their answer with changing views. This was considered no problem, because inconclusive items, items that are not named correctly in the control condition of the task, are excluded in the scoring procedure of the L94.

To classify the scores we used the reference data published in a manual (Stiers et al., 2000).

#### Motion Perception

Motion perception was assessed with three different computerized motion perception tasks: global motion, motion-defined form, and motion speed. The preliminary cut-off values for the 10^th^ percentile (Van der Zee et al., 2019), as presented in Table 1, are used to classify the participant’s performance.

**TABLE 1:** Tenth percentile scores for global motion, motion speed and motion defined-form task in different age groups.

	Global motion (GM)		Motion-defined form (MDF)		Motion speed (MS)	
	*n*	Coherence level	*n*	Proportion correct	*n*	Speed difference (deg/s)
4y3m–4y7m	31	0.78	31	0.45	25	23.80
4y9m–5y8m	39	0.69	43	0.63	34	20.00
5y10m–7y4m	45	0.46	43	0.74	31	12.49

All stimuli consisted of white dots on a black background, with a resolution of 640 × 480 and refresh rate 25 frames/s. In the global motion task and the motion speed task psychophysical thresholds were estimated by calculating the mean of the values of the last 4 of 8 reversals, using a 2 up-1 down staircase procedure. In these tasks, a correct answer was followed by a beep. Before each task, example stimuli were used to familiarize participants with task elements and verify that they understood the task.

##### Global Motion

**FIGURE 1:**
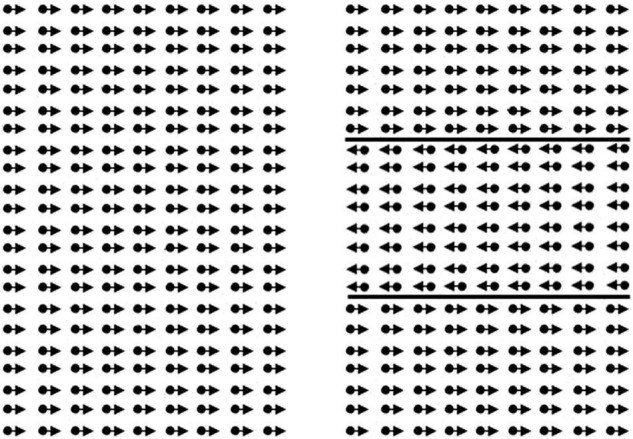
Illustration of the global motion task. The child had to point out the right side. In the real task, lines of the rectangle to the right are not present.

The global motion stimulus (Figure 1) consisted of two random dot kinematograms (RDK size 14.7 × 22.4 deg) containing 1,103 white dots (dot size 0.07 deg, limited lifetime 130 ms), presented next to one another with a distance between them (size 3.3 deg). A variable proportion of dots (starting level 100%, scaling factor 0.33) in each RDK oscillated coherently in horizontal direction (reversal time 330 ms, velocity 6.7 deg/s). Participants had to locate a horizontal strip (size 14.7 × 7.5 deg) in the middle of one of the RDKs, where the coherent dots oscillated in the opposite direction. Participants were instructed to help a lost person to find his way in the snow (presentation = 15 s, answer time 5 s). Because the proportion of coherent dots was constant throughout the RDKs, the strip could not be located by tracing the movement of single dots. The proportion of coherently moving dots, or the coherence level determined the difficulty of the task and was used to calculate the coherence threshold.

##### Motion-Defined Form

**FIGURE 2:**
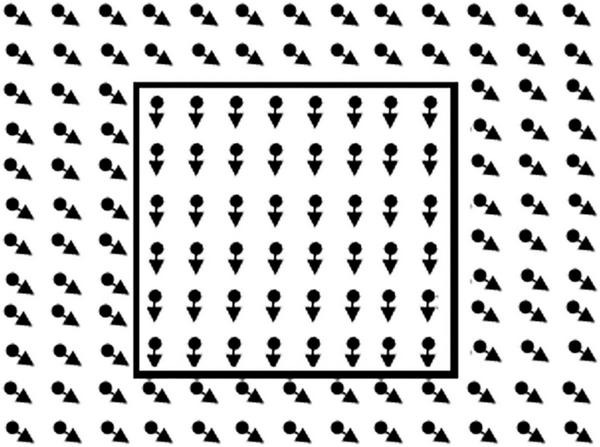
Illustration of the global motion task. In the real task, lines of the rectangle to the right are not present. The child had to point out the right side.

The motion-defined form stimuli (Figure 2) consisted of objects hidden in a RDK (size 20.6 × 16.0 deg, 5,000 dots, dot size 0.13 deg, lifetime 200 ms, velocity 3.4 deg/s). Each object could be displayed in three successive conditions with decreasing level of difficulty (presentation max. 15 s). In all conditions, the dots outside the contour moved coherently in oblique direction. In the first condition, the dots in the contour of the object moved coherently downwards. In the second condition, the dots in the contour were standing still, and in the third condition there were no dots in the contour. After an object was correctly identified the trial was aborted and the next trial, with a new object, was started. If the object was correctly named or described in the first, second or third condition a score of 1, 0.5, or 0 was noted. If the object was not correctly identified in the third condition the response was marked as inconclusive, and the item was not used in the computation of the visual motion perception score. Three subtasks, increasing in difficulty, with six objects were presented. Objects in task 1 were: circle, star, bear, banana, heart, and fish; task 2: Arrow, kangaroo, boat, guitar, ostrich, and bag; task 3: beetle, seat, airplane, seahorse, car, and shoe.

##### Motion Speed

**FIGURE 3:**
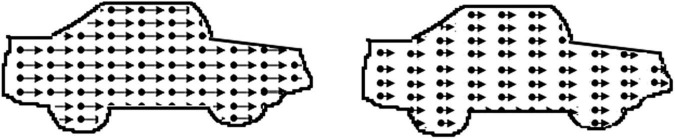
Illustration of the motion speed task: dots in the left car move faster. In the real task, the contour of the car is not present.

The motion speed stimulus (Figure 3) consisted of two identical contours of a car (car length approx. 17 deg) filled with leftwards moving dots (dot density 11 dots/deg^2^, dot size 0.07 deg, lifetime 120 ms). Participants were asked to indicate the location of the fastest car (presentation time 10 s). A decrease in the speed difference of the dots in the cars made the task more difficult (starting speed difference 17.0 deg/s, scaling factor 0.33, 0.25 from fifth reversal) and the critical speed difference was the score for this task.

#### Visual Attention

**FIGURE 4:**
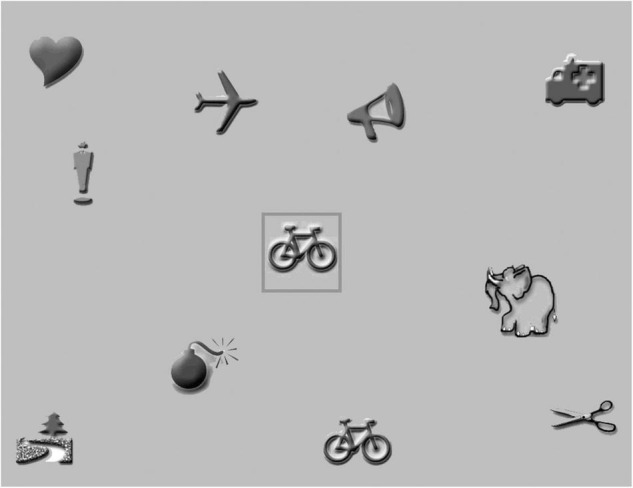
Example of the visual search task with nine distracters and 1 target (bike).

Visual attention was assessed by a computerized visual search task, developed at the department of Psychologic and Pedagogic Sciences of Leuven Catholic University. It consists of greyscale pictures (size 5 × 5 degrees at 40 cm distance) on a gray background (area size approximately 37 × 29 degrees, see example in Figure 4).

Before testing was started, all pictures were presented on the screen to familiarize the participants with the pictures. The participants were asked to name the individual pictures, after which the red-bordered target picture in the center of the screen was introduced. The participant was instructed to point out the picture identical to the central picture as fast and accurate as possible and to put his/her hands upon the table in front of the screen before each trial. The moment the participants touched a picture on the screen, the test administrator pressed the spacebar, and the trial was ended. To verify that the participants understood the task, three practice trials with a target stimulus and two distracters were presented.

Testing was started with a simple reaction-time task with five trials. In the reaction-time task only the central picture and the target picture were presented. Because the location of the target stimulus would pop-out to the participants, the reaction time was considered identical to the motor response time. The motor response time not only included the time needed by the participants to point out the target stimulus, but also the time needed by the administrator to press the spacebar.

In the next stage, three visual search tasks were presented with four, nine, and nineteen distracters. Each task consisted of 10 trials. If needed, the participant was encouraged to keep looking. To control for effects of fatigue and task experience, testing was ended with the above reaction time task.

All reaction times were saved, and the administrator noted all false alarms (mistakes: child pointed out a wrong picture). Reaction times for false alarms were excluded. Median reaction times were calculated for both reaction time tasks and for each visual search task. Because the presence of distracters in the search tasks made pop-out less likely, a serial search process (scanning individual pictures) was assumed to be needed to detect the targets. In a serial search process, the reaction time is the sum of the motor response time and the visual search time. Visual search time was our primary outcome measure, so median reaction times of the reaction time tasks were distracted from median reaction times of the search tasks.

We used the preliminary reference data of 60 typically developing children (25 boys, 35 girls) without any signs of neurological or visual impairment and with a normal visual acuity. They were recruited through primary schools in Rotterdam, The Netherlands. Their chronological age ranged from 4y3 m to 7y4m (*M* = 5y7m *SD* = 9 m). We divided the group in three age groups, equal to those for the motion perception tasks. Cut-off values for the 10^th^ percentile can be found in Table 2.

**TABLE 2:** Tenth percentile scores for visual search outcomes in different age groups.

		5 Items	10 Items	20 Items	Reaction task	Overall
	*n*	Search time (s)	Search time (s)	Search time (s)	Respon se time (s)	Total number of errors
4y3m–4y7m	7	1.38	2.75	5.91	2.54	2
4y9m–5y8m	23	1.23	2.20	5.47	2.03	1
5y10m–7y4m	26	1.09	2.66	4.16	1.70	1

#### Visuomotor Skills

To assess visuomotor skills, we administered the Beery-Buktenica Developmental Test of Visual-Motor Integration (Beery VMI) (Beery et al., 2004) and the subtest Mosaics of the SON-R 2 1/2–7 (Tellegen et al., 1998) as prescribed in the manuals.

##### Beery VMI

Each participant was asked to imitate and copy a maximum of 24 items of increasing complexity. The participant had one try per item. Tracing the pattern and erasing was not allowed. The scoring instructions in the manual were used to decide whether the copy was correct or incorrect. The test was discontinued after 3 consecutive failures (Beery et al., 2004).

##### Mosaics

In Mosaics the participants must copy a maximum of 15 mosaic patterns in a frame. The difficulty level was determined by whether the examiner demonstrates the item, the number and color (red, yellow, and/or red/yellow) of squares the participant must use and the scale of the printed pattern. After each item feedback was given whether the solution was correct or incorrect. In case an incorrect solution was given, the participant was engaged in correcting the solution, without explaining why the solution was incorrect. The subtest was discontinued after a total of three mistakes or two consecutive mistakes at the advanced level (Tellegen et al., 1998).

### Analysis

Participants were included in the analysis if at least three out of five L94 tasks had been completed.

An assessment with 5 tasks, instead of 1, increases the chance of finding at least one abnormally low score. To be confident that our patient group is at risk for object recognition problems, the percentage abnormally low scores should exceed the base rate. The base rate is the expected percentage of the healthy population that would show 1 or more abnormally low test-scores (<5^th^ percentile, *z* = −1.645) on the battery of the five object recognition tasks. To estimate the base rate we used the correlations between the object recognition tasks in the reference group and then performed the Monte Carlo simulation method described by Crawford et al. (2007).

Then, we used the estimated base rate as a fixed number and used the one-sample binomial test to decide whether the observed percentage of abnormally low scores indeed exceeded the base rate. A one-sided alpha ≤ 0.05 was considered significant.

Next, we studied whether children with object recognition problems (score < 5^th^ percentile on L94) had an increased risk of a general dorsal stream dysfunction. A dysfunction or weak function was considered present if a patient showed a score below the 10^th^ percentile (*z* = –1.282), in one of the other dorsal stream tasks. A patient was considered to have a general dorsal stream dysfunction if that patient showed at least two weak functions, i.e., had at least one abnormally low score in two out of three other dorsal stream functions: motion perception, visual attention and/or visuomotor skills. We therefore only included patients who completed at least one task per function. If only the group with object recognition problems was at risk for a general dorsal stream dysfunction, the percentage of at least 2 weak functions in this group had to be significantly higher than the base rate and the percentage of at least 2 weak functions in the patient group without object recognition problems (no score < 5^th^ percentile on L94).

We used the Monte Carlo simulation method to calculate the base rate for at least 1 abnormally low score per function and then calculated the base rate for at least 2 weak functions. We used this estimated base rate as fixed numbers and performed the binomial test to decide whether the observed percentage exceeded this base rate.

To decide whether the percentages of weak functions and scores below the 10^th^ percentile in the group with object recognition problems exceeded those found in the group without object recognition problems we used the Fisher’s exact test. This test was chosen, because of the smaller sample sizes, non-normal data and expected low counts (<5) (Sullivan et al., 2016). Additionally, we used the Mann-Whitney *U*-test to decide whether the performance levels in the group with object recognition problems were worse than in the group without object recognition problems. A one-sided alpha ≤ 0.05 was considered significant.

## Results

### Medical History and Developmental Age

Eight patients out of 48 patients (17%) had low vision, a best corrected decimal visual acuity between 0.1 and 0.3 (US notation 20/200–20/63 or 1.0 logMAR–0.5 logMAR), but all patients should be able to perceive the detailed stimuli. Their developmental age, the median age equivalent of non-verbal intelligence subtests [as described in the procedure by Stiers et al. (1999)], ranged from 2y5 m to 7y8 m (*M* = 5y3 m, *SD* = 1y5 m). Nineteen patients (40%) had been born prematurely (gestational age < 37 weeks): 6 moderately to late premature (gestational age 32–37 weeks), 12 very premature (gestational age 28–32 weeks) and 1 extremely premature (gestational age < 28 weeks). In 31 patients (65%) a motor disorder was present. In another 3 patients (6%) some motor developmental problems were suspected, because a delay in motor development was mentioned in their medical record. More patient characteristics are presented in Tables 3, 4.

**TABLE 3:** Presence of neurodevelopmental conditions in patients with confirmed or suspected brain damage.

Neurodevelopmental conditions	Patient group (*n* = 48)	
	*n*	%
**Etiology**		
Asphyxia	5	10
Hypoxic-ischemic encephalopathy (HIE)		
Periventricular leukomalacia (PVL)	18	38
Intraventricular hemorrhage (IVH)	3	6
PVL + IVH	1	2
Malformation	3	6
Hydrocephalus	1	2
Intracranial hemorrhage (ICH)	1	2
Intracranial hemorrhage + cytomegalovirus infection	1	2
Acquired brain damage (8 months–2.5 years)		
Tumor	1	2
Trauma	4	8
Meningitis	1	2
Genetic	3	6
Unclear	6	12
**Neonatal condition**		
Prematurity (Gestational age <37 weeks)	20	41
**Performance IQ (PIQ)**		
Normal IQ (>84)	12	25
Borderline (71–84)	11	23
Mild retardation (50–70)	12	25
Moderate retardation (<50)	6	13
Unknown	7	15
**Motor disorder**	**31**	**65**
Spastic cerebral palsy		
Hemiplegia	7	15
Diplegia	8	16
Quadriplegia	3	6
Undefined	1	2
Non-spastic cerebral palsy		
Athetoid	2	4
Ataxic	1	2
Mixed cerebral palsy	3	6
Bipyramidal syndrome	4	8

**TABLE 4:** Presence of (neuro-) ophthalmologic conditions in patients with confirmed or suspected brain damage.

(Neuro-) ophthalmologic conditions	Patient group (*n* = 48)	
	*n*	%
**Refractive error**	**9**	**19**
Anisohyperopia (difference > 2D)	2	4
Hyperopia (>+1D and <+6D)	3	6
Hyperopia gravior (≥+6D)	2	4
Pseudophakia	2	4
**Retinopathy of prematurity**		
Stage I or II	2	4
**Optic disc abnormality**	**7**	**15**
Pale appearance	4	8
Smaller than normal	1	2
Optic nerve atrophy (posttraumatic)	2	4
**Strabismus**	**16**	**33**
Manifest	10	21
Intermittent	4	8
Latent	2	4
**Oculomotor dysfunction**	**8**	**16**
Nystagmus		
Manifest	3	6
Latent	1	2
Undefined	1	2
Saccadic dysfunction	2	4
Convergence abnormality	1	2
Horizontal oculomotor apraxia	1	2
**Visual field defect**	**13**	**27**
Scotoma	1	2
Mixed (hemi and altitude)	2	4
Hemianopsia	7	15
Altitude defect	1	2
Concentric, one side more affected	2	4
**Other ophthalmologic conditions**	**5**	**10**
Bilateral cataract	2	4
Posterior embryotoxon	1	2
Septo-optic dysplasia (SOD)	1	2
Choroidal coloboma + peripheral fundus abnormality + intact optic nerve	1	2

### L94: Object Recognition

At least three out of the five L94 tasks could be evaluated in 46 of 48 patients: 5 Tasks in 35, 4 in 7, and 3 in 4 patients. No tasks were systematically missing.

Based on the Monte Carlo simulation, 19.6% of the healthy population was expected to show at least 1 abnormally low score (<5^th^ percentile). Two or more abnormally low scores was expected in 4.38% and at least 3 abnormally low scores in 0.87%. The expected percentage for 4 or more abnormally low scores was near zero.

In the patient group the number of abnormally low scores ranged from 0 to 3, with a median of 1 and an interquartile range (IQR) of 1.25.

An abnormally low score on at least 1 L94 task was found in 29 out of 46 patients (63.0%). This was significantly higher than expected in the healthy population (63.0%, 95% CI:47.5–76.8% vs. 19.6%, *z* = 7.18, *p* < 0.01). At least 2 abnormally low scores were found in 11 patients (23.9%), which was significantly higher than the corresponding base rate (23.9%, 95% CI: 12.6–38.3% vs. 4.38%, *z* = 6.11, *p* < 0.01). Of these 11 patients 6 patients (13% of the total patient group) had 3 abnormally low scores, which was also significantly higher than expected (13.0%, 95% CI: 4.9–26.3% vs. 0.87%, *z* = 8.10, *p* < 0.01). These results showed that this patient group might indeed be at risk for dorsal stream dysfunctions.

Analysis on subtask level showed that only 4 out of 41 patients tested with VISM had an abnormally low score (9.8%). This was not significantly higher than was expected on a single task (9.8%, 95% CI: 2.7–23.1% vs. 5%, *z* = 1.04; *p* = 0.15, *ns*). Three of these patients scored abnormally low on at least one other task: DE VOS and/or NOISE. The percentages of abnormally low scores were significantly higher for the other four tasks: 8/45 for NOISE (17.8%, 95% CI: 8.0–32.1% vs. 5%, *z* = 3.59; *p* < 0.01); 6/45 for OVERL (13.3%, 95% CI: 5.1–26.8% vs. 5%, *z* = 2.22; *p* = 0.01); 13/44 for VIEW (29.5%, 95% CI: 16.8–45.2% vs. 5%, *z* = 7.13; *p* < 0.01); 15/40 for DE VOS (37.5%, 95% CI: 22.7–54.2% vs. 5%, *z* = 9.07; *p* < 0.01).

### Dorsal Stream Functioning in Patients With and Without Object Recognition Problems

Out of the remaining 46 patients, 44 completed at least one motion perception task, 41 at least one visuomotor task, and 33 the visual attention task. This resulted in 29 patients in whom both at least three L94 tasks and all three other dorsal stream functions could be evaluated. This group only included 1 patient with an abnormally low score on VISM.

Of these patients 18 patients had object recognition problems and 11 had no object recognition problems. The median number of evaluated tasks of other dorsal stream functions was 5 out of totally 7 tasks (range 4–5, IQR 0). There was no difference between groups in the number of tasks evaluated. The group with and without object recognition problems did neither differ significantly in CA (6y2 m, range 4y1 m–8 y7m, IQR 1y5 m vs. 6y2 m, range 4y1 m–10y2 m, IQR 0y4 m; *U* = 96.5, *z* = –0.11, *p* = 0.91, *ns*) nor in PIQ (69, range 50–117, IQR 41.25 vs. 89, range 57–121, IQR 22; *U* = 57.5, *z* = –1.48, *p* = 0.07, *ns*), nor in DA (5y6 m, range 3y1 m–7y8m, IQR 2y6m vs. 4y8m, range 2y5 m–6 y8m, IQR 2y1 m; *U* = 66, *z* = –1.48, *p* = 0.15, *ns*). In the patient group with object recognition problems 5 had low vision (decimal visual acuity 0.1–0.3, US notation 20/200–20/63 or 1.0 logMAR–0.5 logMAR) and could be considered visually impaired. Five patients had a subnormal visual acuity for their age (decimal visual acuity 0.5–0.8, US notation 20/40–20/25 or 0.3–0.1 logMAR). The remaining 8 patients had a normal visual acuity. In the patient group without object recognition problems 2 had a subnormal visual acuity and the remaining 9 had a normal visual acuity.

#### Risk of a Generalized Dorsal Stream Dysfunction

Based on the Monte Carlo simulation method 27.9% of the healthy population was expected to show at least one abnormally low score (<10^th^ percentile) on the motion perception tasks, 21.1% was expected to show at least at least on abnormally low score on the visual attention task, and 16.8% was expected to show at least one abnormally low score on the visuomotor tasks. Based on these results we expected 11.8% of the population to show at least 2 weak dorsal stream functions and about 1% (0.95%) to show 3 weak dorsal stream functions.

In the group with object recognition problems 6 patients (33.3%) were considered to have at least 2 additional weak dorsal stream functions. This was significantly higher than expected in the healthy population (33.3%, 95% CI: 13.3–59.0% vs. 11.8%, *z* = 2.47; *p* = 0.01). Two patients showed 3 weak dorsal stream functions, which could be considered relatively high (11.1%, 95% CI: 1.4–34.7 vs. 1.0%, *z* = 3.13; *p* = 0.01).

In the group with normal object recognition 1 patient (9.1%) had at least 2 weak dorsal stream functions. This was as expected in the healthy population (9.1%, 95 % CI: 0.0–41.3% vs. 11.8%, *z* = 0; *p* = 0.50. *ns*).

The observed percentages of at least 2 weak dorsal stream functions in the group with and without object recognition problems did not differ significantly [difference 24.1%, *p* = 0.20 (two-sided), *p* = 0.15 (one-sided)], probably due to the small sample sizes. A *post hoc* power-analysis (power of 80%, confidence level of 95%) suggested that future samples should at least have 41 participants per group.

These results suggested that general dorsal stream dysfunctions might be more common in the group with object recognition, but our evidence is not strong enough. Additional analysis suggested that dorsal stream dysfunctions, specific and general, were more common in the patient group with object recognition problems: 13 out of 18 patients with object recognition problems (72.2%) had at least 1 weak function, while only 3 out of 11 patients without object recognition problems (27.3%) had at least 1 weak function (*Fischer’s exact one-sided p-value* = *0.02)*. Most of the patient group with object recognition problems had other dorsal stream problems: 6 were considered to have a general dorsal stream dysfunction and an additional 7 were considered to have a specific dorsal stream dysfunction. In the patient group without object recognition problems 1 was considered to have a general dorsal stream dysfunction and 2 were considered to have a specific dorsal stream dysfunction.

#### Outcomes of Different Dorsal Stream Aspects

In Tables 5–7, we present number of abnormally low scores per function and (sub) task and median task outcomes for both groups.

**TABLE 5:** Number of abnormally low scores (<10^th^ percentile) for motion perception subtasks and subtask outcomes for patient with normal and abnormally low scores on the L94 (<5^th^ percentile).

	Patients with 3 additional function evaluations		Statistics			
	Normal L94	≥ 1 Abnormal L94 tasks	*U*	*z*	Two-sided *p*-value	One-sided *p*-value^a^
		
	*n* = 11	*n* = 18				
**Motion perception**						
# Abnormal scores (%)	1/11 (9.1)	9/18 (50.0)			0.04*	0.03*
**Global motion task (GM)**						
# Abnormal scores (%)	0/10 (0.0)	7/16 (43.8)			0.02*	0.02*
Median coherence level (range) (IQR)	0.42 (0.32–0.58) (0.11)	0.53 (0.12–0.83) (0.43)	47.5	–1.71	0.09	0.04*
**Motion-defined form task (MDF)**						
# Abnormal scores (%)	1/10 (10)	5/16 (31.3)			0.35	0.23
Median percentage correct (range) (IQR)	92 (73–1.00) (16)	71 (0– 97) (27)	25.5	–2.88	<0.01*	<0.01*
**Motion speed task (MS)**						
# Abnormal scores (%)	0/9 (0.0)	0/5 (0.0)			*NA*	*NA*
Median speed difference deg/s (range) (IQR)	4.83 (1.70–9.66) (5.66)	4.28 (1.27–23.24) (12.01)	22.0	–0.07	1.0	0.50

*^a^We used the one-sided p-values, because we expected the patients with object recognition problems to perform worse.*

*Significant at significance level 0.05; NA, not available, values are equal/constant.

##### Motion Perception

The group with object recognition problems had a significantly higher percentage of abnormally low scores on motion perception (50% vs. 9.1%; *p* = 0.03) than the group without object recognition problems (see Table 5). Abnormally low scores were found in a single motion perception task (GM or MDF) in 6 patients with and 1 patient without object recognition problems. Another 3 patients with object recognition problems had abnormally low scores on GM and MDF.

The group with object recognition problems scored significantly more often abnormally low on GM (*p* = 0.02) and their coherence level was significantly higher than that in patients without object recognition problems (coherence level 0.53 vs. 0.42; *p* = 0.04). Although the percentage of abnormally low scores did not differ significantly on MDF, patients with object recognition problems were significantly less able to recognize the motion-defined forms (percentage correct 72% vs. 92%, *p* < 0.01). No significant differences were found for the motion speed task.

##### Visual Attention

The percentage of abnormally low scores on the visual attention task (see Table 6) was significantly higher in the group with object recognition problems (*p* = 0.03).

**TABLE 6:** Number of abnormally low scores (<10^th^ percentile) for the visual attention task and subtask outcomes for patient with normal and abnormally low scores on the L94 (<5^th^ percentile).

	Patients with 3 additional function evaluations		Statistics			
	Normal L94	≥ 1 Abnormal L94 tasks	*U*	*z*	*Two-sided p*-value	*One-sided p-value^a^*
		
	*n* = 11	*n* = 18				
**Visual attention**						
# Abnormal scores (%)	1/11 (9.1)	9/18 (50)			0.04*	0.03*
**Search task 4 distracters**						
# Abnormal scores (%)	1/11 (9.1)	7/18 (38.9)			0.11	0.09
Median search time sec (range) (IQR)	0.73 (0.00–1.97) (0.41)	1.21 (0.56–3.45) (1.09)	48.00	–2.29	0.02*	0.01*
**Search task 9 distracters**						
# Abnormal scores (%)	0/11 (0)	3/18 (16.7)			0.27	0.22
Median search time sec (range) (IQR)	1.13 (0.72–1.71) (0.46)	1.77 (0.71–6.72) (1.59)	33.5	–2.95	<0.01*	<0.01*
**Search task 19 distracters**						
# Abnormal scores (%)	0/11 (0)	3/18 (16.7)			0.27	0.22
Median search time sec (range) (IQR)	2.11 (0.99–3.46) (1.55)	3.90 (1.17–11.55) (3.35)	36.00	–2.83	<0.01*	<0.01*
**Mistakes**						
# Abnormal scores (%)	0/11 (0)	9/18 (50)			<0.01*	<0.01*
Median number (range) (IQR)	0 (0–0) (0)	1 (0–11) (3)	27.50	–3.54	<0.01*	<0.01*
**Reaction time task**						
# Abnormal scores (%)	0/11 (0)	1/18 (5.6%)			1.0	0.62
Median response time sec (range) (IQR)	1.32 (0.90–2.55) (0.86)	1.49 (0.94–2.83) (0.83)	76.50	–1.01	0.32	0.16

*^a^We used the one-sided p-values because we expected the patients with object recognition problems to perform worse.*

*Significant at significance level 0.05; NA, not available, values are equal/constant.

Abnormally low scores were mainly found on a single subtask: in 6 patients with and in 1 patient without object recognition problems. In the patient group with object recognition problems another 2 scored abnormally low on 2 subtasks and 1 on all three.

Although the observed percentages of abnormally low scores in the visual search subtasks did not differ significantly. the difference in the condition with four distracters was near-significant (*p* = 0.09). Additionally, patients with object recognition problems were significantly slower on each subtask and made significantly more mistakes than the group without object recognition problems (*p*s < 0.01), whereas the performance on the reaction time task seemed comparable.

##### Visuomotor Skills

No significant differences were found in the visuomotor skills (see Table 7). Abnormally low scores on both tasks were only found in 1 patient with object recognition problems. The other patients seemed to have a specific problem with one of the tasks.

**TABLE 7:** Number of abnormally low scores (<10^th^ percentile) for visuomotor skills and subtask outcomes for patient with normal and abnormally low scores on the L94 (<5^th^ percentile).

	Patients with 3 additional function evaluations		Statistics			
	Normal L94	≥1 Abnormal L94 tasks	*U*	*z*	Two-sided *p*-value	One-sided *p*-value^a^
		
	*n* = 11	*n* = 18				
**Visuomotor skills**						
# Abnormal scores (%)	2/11 (18.2)	3/18 (16.7)			1.0	0.64
**Beery before VMI**						
# Abnormal scores (%)	0/6 (0)	2/16 (12.5)			1.0	0.52
Median standard score (range) (IQR)	96.5 (83–106) (17)	96.0 (73–142) (22)	48.0	0.00	1.0	0.51
**Mosaics (SON-R)**						
# Abnormal scores (%)	2/11 (18.2)	2/18 (11.1)			0.62	0.49
Median standard score (range) (IQR)	10.0 (3–12) (4)	10.5 (5–15) (3.5)	72.5	–1.20	0.24	0.12

*^a^We used the one-sided p-values because we expected the patients with object recognition problems to perform worse.*

*NA = not available, values are equal/constant.*

## Discussion

In this study, we provided some evidence that children with early brain damage are at risk for dorsal stream dysfunctions. While controlling for the patient’s developmental age, 29 out of 46 patients with early brain damage (63%) scored abnormally low (score < 5^th^ percentile) on one or multiple object recognition subtasks of the L94. This was significantly higher than the base rate of 12%, the expected percentage of abnormally low scores in the healthy population.

We then studied whether general dorsal stream problems were present in children with impaired object recognition (*n* = 18) and compared their performance levels to that of children with early brain damage with unimpaired object recognition (*n* = 11). We defined a general dorsal stream dysfunction as abnormally low performance levels (score below 10^th^ percentile) on at least 2 additional dorsal stream functions, such as motion perception, visual attention, and/or visuomotor skills. The results showed that a general dorsal stream dysfunction was present in 6 out of 18 patients with impaired object recognition (33.3%). Another 7 patients scored abnormally low on 1 additional dorsal stream function. Dorsal stream problems were uncommon in patients without object recognition problems: they were only found in 3 out of 11 patients (27.3%). Of these patients only 1 (9.1%) was considered to have a general dorsal stream dysfunction. This was as many as could we expected in the healthy population.

Although these results suggest that patients with object recognition problems are at risk for general, widespread dorsal stream dysfunctions, the difference of 24.1% between the patients with and without object recognition problems was not significant. The higher percentage of patients at least 1 dorsal weakness (72.2% vs. 27.3%, *p* = 0.02) indicates that patients with object recognition problems are at risk of other dorsal stream dysfunctions, possibly rather specific than general. Motion perception and visual attention, but no visuomotor skills, were specifically affected.

In neuropsychology the terms specific and generalized are frequently used, but the definition of generalized remains arbitrary. A specific or independent disorder is considered present if a single aspect is impaired in one patient (group), whereas another aspect is impaired in another patient (group). The term generalized is used when impairments are widespread within and/or across various aspects of functioning. Although in some of our patients the dorsal stream dysfunctions seemed widespread, the dysfunctions within a single aspect, like motion perception seemed rather specific than generalized. Our estimation of a general dysfunction within aspects might be underestimated, because of missing data. The extent of generalized dysfunctions within aspects can only be reliably estimated if all patients were assessed with all subtasks.

In current study we can only deduce that the dorsal stream is affected. We have no details on the exact locations and extent of the brain damage. Although the subtasks of the L94 are likely to activate both the ventral and the dorsal stream, the subtask profile of the L94 suggest that the ventral stream is mainly intact in our patient group. The performance level on VISM, a subtask with line drawings of everyday objects in prototypical view, probably mainly relies on the integrity of the ventral stream, as demonstrated in a fMRI study with passive object viewing (Stiers et al., 2006) and patient studies (Vaina, 1994; Stiers et al., 2001). In our patient group only 4 out of 41 patients (9.8%) scored abnormally low on this subtask. The performance levels in the other four subtasks (OVERL, NOISE, VIEW, and DEVOS), probably rely in a higher extent on the integrity of the dorsal stream due to the suboptimal representations used (Renzi and Spinnler, 1966; Warrington and James, 1967; Warrington and Taylor, 1973; Vaina, 1994; Fazzi et al., 2004; Sheth and Young, 2016). In our patient group we mainly found abnormalities on these other four tasks (range 13.3% in OVERLAP to 37.5% in DE VOS). An abnormality on VISM was almost always accompanied by an abnormality on one of the other tasks. This suggests that mainly the dorsal stream was affected in our patient group, and that in some patient the ventral stream might also be deficient.

Patients with object recognition problems performed worse on the global motion and motion-defined form task and were significantly slower on the visual search tasks. They also made more mistakes, which makes a speed-accuracy trade-off less likely. The available data are insufficient to study causal or interactional relationships between functions. One possibility is that a visual attention weakness leads to abnormally low motion and object perception scores, but performance levels on the assessed tasks could also be low because of difficulties in object discrimination, impulse control, and cognitive flexibility. Therefore, to test hierarchical models, not only a larger sample size but also assessment of additional indicators is necessary.

The presence of object recognition and motion perception problems in combination with the normal visuomotor skill on the Beery VMI support the more fundamental idea that developmental age estimations based on PIQ subtests can be used to control for effects of motor impairments in addition to intellectual impairments. Because the Beery VMI (Beery et al., 2004) can provide age equivalents, it might be suggested that the outcome of the Beery VMI could be used to estimate developmental age and control for motor impairments by using these age equivalents as entry of the norm tables. Although performance IQ and the outcome of the Beery VMI are significantly related (Beery et al., 2004), we consider the developmental age estimation based on PIQ subtests more reliable, because the estimation is based on multiple subtest results instead of a single test outcome. In current study, we found two abnormal performers on the Beery VMI. The use of the age equivalents of the Beery VMI would make this impossible.

Further, a more detailed analysis of inconclusive items, i.e., items that were not named correctly in the control conditions of the L94 and the motion-defined form task and were excluded from the calculation of perception scores, might help explain performance patterns in children at risk. Inconclusive items can indicate differences in experience or object knowledge, but also the presence of other problems such as problems in language development, naming, especially in relation to words (items) with lower word frequencies, memory and attention, or a combination of these problems. Differences in the number of inconclusive items between a group with unimpaired and impaired object recognition could provide indications for the reason why abnormal performers were unable to name or describe objects in the control items.

We conclude that in children with early brain damage, dorsal stream functions and their aspects seem specifically and not generally affected, and that more extensive research is required for a better understanding of causal relationships and underlying mechanisms. For now, we recommend that in specialized clinical practice, multiple functions and their various aspects should be assessed in a neuropsychological assessment of at-risk children, using developmental age as reference level, i.e., entry of the reference table.

## Data Availability Statement

The raw data supporting the conclusions of this article will be made available by the authors, without undue reservation.

## Ethics Statement

The studies involving human participants were reviewed and approved by the Ethics Committees of the Erasmus Medical Centre MEC-2006-056) and the Catholic University of Leuven. Written informed consent to participate in this study was provided by the participants’ legal guardian/next of kin.

## Author Contributions

PS and HE conceived the study. PS and YZ designed the study design and procedures, significantly contributed to procedures and execution, and interpreted the data. YZ, PS, and HE drafted, revised, and prepared the manuscript. All authors gave final approval for the submitted manuscript.

## Conflict of Interest

The authors declare that the research was conducted in the absence of any commercial or financial relationships that could be construed as a potential conflict of interest.

## Publisher’s Note

All claims expressed in this article are solely those of the authors and do not necessarily represent those of their affiliated organizations, or those of the publisher, the editors and the reviewers. Any product that may be evaluated in this article, or claim that may be made by its manufacturer, is not guaranteed or endorsed by the publisher.

## Abbreviations

CA: chronological age
DA: development age, median age-equivalent based on PIQ outcomes
PIQ: Performance Intelligence Quotient
RDK: Random dot kinematogram.
